# The epidemiology of anaphylaxis in Europe: protocol for a systematic review

**DOI:** 10.1186/2045-7022-3-9

**Published:** 2013-03-28

**Authors:** Sukhmeet S Panesar, Bright I Nwaru, Lennart Hickstein, Tamara Rader, Hala Hamadah, Dana Fawzi Ibrahim Ali, Bhavesh Patel, Antonella Muraro, Graham Roberts, Margitta Worm, Aziz Sheikh

**Affiliations:** 1 University of Edinburgh, Teviot Place, Edinburgh, EH8 9AG, UK; 2 University of Tampere, Kalevantie 4, Tampere, FI-33014, Finland; 3 Ludwig-Maximilian-University, Leopoldstr. 3 /018a 80802, Munich, Germany; 4 University of Ottawa, 75 Laurier Avenue East, Ottawa, ON, K1N 6N5, Canada; 5 St. George’s University, Cranmer Terrace, London, SW17 0RE, UK; 6 Open University, Walton Hall, Milton Keynes, Buckingahmshire, MK7 6AA, UK; 7 Padua General University Hospital, Via Giustiniani 3, Padua, 35128, Italy; 8 Faculty of Medicine, University of Southampton, Southampton, SO171BJ, UK; 9 Charité University Charitestraße 1, Berlin, 10117, Germany; 10 Centre for Population Health Sciences, The University of Edinburgh Medical School, Doorway 3, Teviot Place, Edinburgh, EH8 9AG, UK

**Keywords:** Anaphylaxis, Allergy, Epidemiology, Prevalence, Incidence

## Abstract

**Background:**

The European Academy of Allergy and Clinical Immunology is in the process of developing its *Guideline for Food Allergy and Anaphylaxis*, and this systematic review is one of seven inter-linked evidence syntheses that are being undertaken in order to provide a state-of-the-art synopsis of the current evidence base in relation to epidemiology, prevention, diagnosis and clinical management and impact on quality of life, which will be used to inform clinical recommendations.

The aims of this systematic review will be to understand and describe the epidemiology of anaphylaxis, i.e. frequency, risk factors and outcomes of anaphylaxis, and describe how these characteristics vary by person, place and time.

**Methods:**

A highly sensitive search strategy has been designed to retrieve all articles combining the concepts of anaphylaxis and epidemiology from electronic bibliographic databases.

**Discussion:**

This review will aim to provide some estimates of the incidence and prevalence of anaphylaxis in Europe. The occurrence of anaphylaxis can have a profound effect on the quality of life of the sufferer and their family. Estimates of disease frequency will help us to ascertain the burden of anaphylaxis and provide useful comparators for management strategies.

## Background

Anaphylaxis is a ‘severe, life-threatening generalised or systemic hypersensitivity reaction’ [1,2]. Several working definitions of anaphylaxis have been formulated to aid clinical diagnosis and management [3-6]. The most well-known of these is the consensus clinical definition proposed by Sampson et al., which involved representatives of a number of international allergy organisations, including the European Academy of Allergy and Clinical Immunology (EAACI) [7].

Whilst useful clinically, definitions in which the presumption of exposure to a known trigger substantially increase the likelihood of making the diagnosis are of limited value for epidemiological investigations. Hence, current estimates of the epidemiology of anaphylaxis will be subject to uncertainty depending on the case definition used [8]. Reliably establishing the epidemiology of anaphylaxis is further complicated by the fact that it is a relatively uncommon condition, which is acute in onset and transient, rendering it difficult to mount prospective investigations [9]. Investigators may therefore need to use less than ideal study designs, which further results in the possibility of generating biased estimates.

Notwithstanding these inherent challenges, there is a need to improve our understanding of the epidemiology of anaphylaxis in order to inform deliberations on, amongst other things, the overall disease burden posed by the condition, obtain etiological insights, risk stratification and prognosis.

Epidemiological measures of particular interest for anaphylaxis therefore include measures of incidence and prevalence, risk factors, and risk of recurrence and death. The following epidemiological definitions proposed by Last, and adapted for anaphylaxis will be employed in this review: [10].

### Incidence

The number of new cases of anaphylaxis that occur during a given period in a defined population. Incidence will be studied as:

• *Incidence rate:* The number of new cases of anaphylaxis that occur during a defined period per unit person-time.

• *Cumulative incidence*: The number of new cases of anaphylaxis that occur during a given period per the population at risk.

### Prevalence

The proportion of a defined population known to have experienced anaphylaxis. Care is required in defining the appropriate denominator. This epidemiological measure will be further divided into:

• *Point prevalence:* the proportion of the population that has experienced anaphylaxis at a specific time.

• *Period prevalence:* the proportion of the population that has experienced anaphylaxis during a given period.

• *Lifetime prevalence:* the proportion of the population that at some point in their life will have experienced anaphylaxis.

### Case fatality rate

The proportion of cases of anaphylaxis that proves fatal (usually defined within a time period). This is also sometimes known as the case fatality ratio.

Other aspects of interest concern features of persons who experience anaphylaxis, temporal relationships, and the factors that lead to its development and recurrence.

The EAACI is in the process of developing the *EAACI Guideline for Food Allergy and Anaphylaxis*, and this systematic review is one of seven inter-linked evidence syntheses that are being undertaken in order to provide a state-of-the-art synopsis of the current evidence base in relation to epidemiology, prevention, diagnosis and clinical management and impact on quality of life, which will be used to inform clinical recommendations.

## Aims

The aims of this systematic review will be to:

• Understand and describe the epidemiology of anaphylaxis, i.e. frequency, risk factors and outcomes of anaphylaxis

• Describe how these characteristics vary by person, place and time.

## Methods

### Search strategy

**Figure 1:**
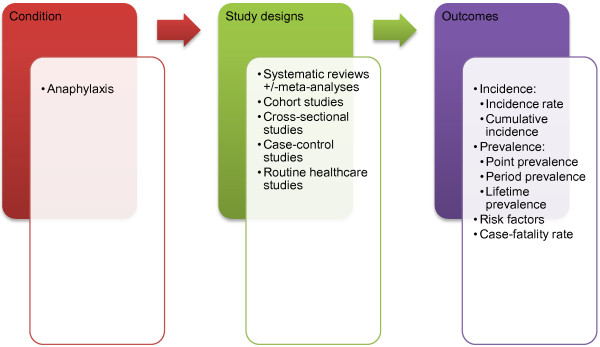
Conceptualisation of systematic review on the epidemiology of anaphylaxis.

A highly sensitive search strategy has been designed to retrieve all articles combining the concepts of anaphylaxis and epidemiology from electronic bibliographic databases. We have conceptualised the search to incorporate three elements, as shown in Figure 1: Conceptualisation of systematic review of the epidemiology of anaphylaxis.

To retrieve systematic reviews, we will use the systematic review filter developed at McMaster University Health Information Research Unit [11]. We have also adapted the search filter from York University Centre for Reviews and Dissemination [12] to retrieve incidence, prevalence and other characteristics describing the epidemiology of anaphylaxis. Similarly, we also applied the McMaster filter for prognosis studies [13].

The following databases will be searched:

• MEDLINE (OVID)

• Embase (OVID)

• CINAHL (Ebscohost)

• ISI Web of Science (Thomson Web of Knowledge).

The search strategy has been devised on OVID MEDLINE and then adapted for the other databases (see Additional file 1). In all cases the databases will be searched from 1 January 2000 to 30 September 2012, and will be limited to Europe based on the definition provided by the Organization for Economic Co-operation and Development (OECD) [14]. The countries covered by this restriction include Austria, Belgium, Czech Republic, Denmark, Estonia, Finland, France, Germany, Greece, Hungary, Iceland, Ireland, Italy, Luxembourg, the Netherlands, Norway, Poland, Portugal, Slovak Republic, Slovenia, Spain, Sweden, Switzerland, Turkey and the United Kingdom. All references will be imported into an EndNote Library and tagged with the name of the database. Searches will be limited to literature from 2000 onwards; because we want to understand and describe the contemporary epidemiology of anaphylaxis.

Additional references will be located through searching the references cited by the identified studies, and unpublished work and research in progress will be identified through discussion with experts in the field. We will invite experts who are active in the field from a range of disciplines and geography to comment on our search strategy, and the list of included studies.

There will be no language restrictions and, where possible, all literature will be translated.

### Inclusion criteria for study design

• Systematic reviews +/-meta-analyses

• Cohort studies

• Cross-sectional studies

• Case-control studies

• Routine healthcare studies

These study designs were chosen to ensure that the highest levels of evidence were pooled based on the aims of this review [15].

### Exclusion criteria for study design

• Reviews, discussion papers, non-research letters and editorials

• Case studies and case series

• Animal studies

### Study selection

The titles of the retrieved articles will be checked independently by two reviewers according to the above selection criteria and categorised as: included, not included and unsure. For those papers in the unsure category we will retrieve the abstract and re-categorise as above after further discussion on them. Any discrepancies will be resolved by consensus and if necessary a third reviewer will be consulted to arbitrate. Full text copies of potentially relevant studies will be obtained and their eligibility for inclusion independently assessed by two reviewers. Studies that do not fulfil all of the inclusion criteria will be excluded.

### Risk of bias assessment strategy

Risk of bias assessments will be independently carried out on each study by two reviewers using the relevant version of the Critical Appraisal Skills Programme (CASP) quality assessment tool for systematic reviews [16], cohort studies [17] and case-control studies [18], which involves an assessment of both internal and external validity [19]. Similarly, we will use the Effective Public Health Practice Project Quality Assessment Tool (EPHPP) for assessing other forms of quantitative studies such as cohort analyses, cross-sectional studies and routine healthcare studies [20]. An overall grading and grading for the various components of each study (e.g. the appropriateness of the study design for the research question, the risk of selection bias, exposure measurement, and outcome assessment) will be given to each study. Any discrepancies will be resolved by discussion or, if agreement could not be reached, by arbitration by a third reviewer.

### Analysis, data synthesis and reporting

Data will be independently extracted onto a customised data extraction sheet by two reviewers, and any discrepancies will be resolved by discussion or, if agreement could not be reached, by arbitration by a third reviewer.

A descriptive summary with data tables will be produced to summarise the literature. If clinically and statistically appropriate, meta-analysis using either fixed-effector random-effects modeling will be undertaken using methods suggested by Agresti and Coul [21]. A narrative synthesis of the data will also be undertaken.

This review has been registered with the International Prospective Register of Systematic Reviews (PROSPERO) and has registration number CRD42013003702 allocated to it. The Preferred Reporting Items for Systematic Reviews and Meta-Analyses (PRISMA) checklist will be used to guide the reporting of the systematic review [22].

## Discussion

The occurrence of anaphylaxis can have a profound effect on the quality of life of the sufferer and their family. The risk of recurrence may be high and some attacks prove fatal, sometimes despite immediate, on-site treatment with epinephrine. Successfully identifying those at greatest risk of an initial attack, and a recurrence, could reduce morbidity, but this has proved difficult in practice using demographic and clinical markers. Secondary analyses of routine sources of data have proved helpful in describing the epidemiology of anaphylaxis though the estimates generated would be considered more reliable if the data could be validated and linked across primary and secondary care sectors. Such validation work needs to be prioritized. This review will aid in providing some estimates of the frequency of anaphylaxis in Europe. At present, the best epidemiological estimates appear to come from North-West Europe, but more information is needed from Southern and Eastern Europe.

## Abbreviations

EAACI: European academy of allergy and clinical immunology; EPHPP: Effective public health practice project quality assessment tool; OECD: Organization for economic co-operation and development; CASP: Critical appraisal skills programme; PROSPERO: Prospective register of systematic reviews; PRISMA: Preferred reporting items for systematic reviews and meta-analyses.

## Competing interests

The authors declare that there are no competing interests, financial or otherwise.

## Authors’ contributions

SSP, BN, LH, TR, HH, DF-IA and BP conceptualised and designed the protocol and drafted earlier versions of the document in their capacity as methodologists. AM, GR and MW contributed to further refinements of the protocol and revised it critically for important intellectual content in their capacity as guideline leads. AS led on the development of concepts used in this protocol and revised it critically for important intellectual content in his capacity as the methodology lead. All authors approved the final version to be published.

## Supplementary Material

Additional file 1 Search strategies. Click here for file

## References

[B1] Johansson SGO Bieber T Dahl R Friedmann PS Lanier B Lockey RF Motala C Ortega Martell JA Platts-Mills TA Ring J Thien F Van Cauwenberge P Williams HC A revised nomenclature for allergy for global use: report of the nomenclature review committee of world allergy organization J Allergy Clin Immunol 2004 113 832 836 10.1016/j.jaci.2003.12.591 15131563

[B2] Muraro A Roberts G Clark A Eigenmann PA Halken S Lack G Moneret-Vautrin A Niggemann B Rancé F EAACI task force on anaphylaxis in children. The management of anaphylaxis in childhood: position paper of the European academy of allergology and clinical immunology Allergy 2007 62 8 857 871 10.1111/j.1398-9995.2007.01421.x 17590200

[B3] American Academy of Pediatrics Committee on school health. Guidelines for urgent care in school Pediatrics 1990 86 999 1000 2251038

[B4] International Collaborative Study of Severe Anaphylaxis An epidemiologic study of severe anaphylactic and anaphylactoid reactions among hospital patients: methods and overall risks Epidemiology 1998 9 141 146 10.1097/00001648-199803000-00007 9504281

[B5] Australasian Society of Clinical Immunology and Allergy Inc (ASCIA) Guidelines for EpiPen prescription. ASCIA Anaphylaxis Working Party 2004 Available online at http://www.allergy.org.au/anaphylaxis/epipen_guidelines.htm Last accessed on 20th September 2012

[B6] Joint Task Force on Practice Parameters; American Academy of Allergy, Asthma and Immunology; American College of Allergy, Asthma and Immunology; and Joint Council of Allergy, Asthma, and Immunology The diagnosis and management of anaphylaxis: an updated practice parameter J Allergy Clin Immunol 2005 115 3 suppl S483 S523 15753926 10.1016/j.jaci.2005.01.010

[B7] Sampson HA Muñoz-Furlong A Campbell RL Adkinson NF Bock A Branum A Brown SG Camargo CA Jr Cydulka R Galli SJ Gidudu J Gruchalla RS Harlor AD Jr Hepner DL Lewis LM Lieberman PL Metcalfe DD O’Connor R Muraro A Rudman A Schmitt C Scherrer D Simons FE Thomas S Wood JP Decker WW Second symposium on the definition and management of anaphylaxis: summary report-second national institute of allergy and infectious disease/food allergy and anaphylaxis network symposium J Allergy Clin Immunol 2006 117 391 397 10.1016/j.jaci.2005.12.1303 16461139

[B8] Lieberman P Camargo CA Bohlke K Jick H Miller RL Sheikh A Simons FE Epidemiology of anaphylaxis: findings of the American college of allergy, asthma and immunology epidemiology of anaphylaxis working group Ann Allergy Asthma Immunol 2006 97 596 602 10.1016/S1081-1206(10)61086-1 17165265

[B9] Simons FE Sheikh A Evidence-based management of anaphylaxis Allergy 2007 62 827 829 10.1111/j.1398-9995.2007.01433.x 17620059

[B10] Last JM A dictionary of epidemiology 2000 4 New York: Oxford University Press

[B11] McMaster university health information research unit http://hiru.mcmaster.ca/hiru/HIRU_Hedges_MEDLINE_Strategies.aspx#Reviews

[B12] York University centre for reviews and dissemination http://www.york.ac.uk/inst/crd/intertasc/epidemiological_studies.html

[B13] McMaster filter for prognosis studies http://hiru.mcmaster.ca/hiru/HIRU_Hedges_EMBASE_Strategies.aspx

[B14] The organization for economic Co-operation and development http://www.oecd.org/about/membersandpartners/

[B15] OCEBM Levels of Evidence Working Group The Oxford 2011 levels of evidence Oxford Centre for Evidence-Based Medicine Available online at http://www.cebm.net/index.aspx?o=5653 Last accessed on 28th September 2012

[B16] CASP checklist for systematic reviews http://www.casp-uk.net/wp-content/uploads/2011/11/CASP_Systematic_Review_Appraisal_Checklist_14oct10.pdf Last accessed on 10th October 2012

[B17] CASP checklist for cohort studies http://www.casp-uk.net/wp-content/uploads/2011/11/CASP_Cohort_Appraisal_Checklist_14oct10.pdf Last accessed on 10th October 2012

[B18] CASP checklist for case-control studies http://www.casp-uk.net/wp-content/uploads/2011/11/CASP_Case-Control_Appraisal_Checklist_14oct10.pdf Last accessed on 10th October 2012

[B19] Appraisal tools http://www.casp-uk.net/ Last accessed on 20th September 2012

[B20] Effective public health practice project quality assessment tool http://www.ephpp.ca/Tools.html Last accessed on 10th October 2012

[B21] Agresti A Coull BA Approximate is better than ‘exact’ for interval estimation of binomial proportions Am Statis 1998 52 119 126

[B22] Moher D Liberati A Tetzlaff J Altman DG The PRISMA group. Preferred reporting items for systematic reviews and meta-analyses: the PRISMA statement PLoS Med 2009 6 7 e1000097 10.1371/journal.pmed.1000097 19621072 PMC2707599

