# Supplementary material for: The epidemiology of anaphylaxis in Europe: protocol for a systematic review
Source: Clin Transl Allergy. 2013 Mar 28;3:9. doi: 10.1186/2045-7022-3-9 (PMC3685580; doi:10.1186/2045-7022-3-9)
Supplement: Additional file 1 — Search strategies. [file 2045-7022-3-9-S1.docx]

**Additional file 1: Search strategies**

*Database: Ovid MEDLINE(R) In-Process & Other Non-Indexed Citations and Ovid MEDLINE(R) <1990 to Present>*

Search Strategy:

--------------------------------------------------------------------------------

| 1 | Anaphylaxis/ |
| --- | --- |
| 2 | anaphylaxis react*.mp. |
| 3 | anaphylactic react*.mp. |
| 4 | anaphylactic shock*.mp. |
| 5 | anaphylactoid syndrome*.mp. |
| 6 | anaphylactoid react*.mp. |
| 7 | anaphylactic syndrome*.mp. |
| 8 | anaphylactoid shock*.mp. |
| 9 | acute systemic allergic react*.mp. |
| 10 | idiopathic anaphylaxis.mp. |
| 11 | systemic anaphylaxis.mp. |
| 12 | or/1-11 |
| 13 | (rat or rats or cow or cows or chicken? or horse or horses or mice or mouse or bovine  or animal$).ti. |
| 14 | exp animals/ not humans.sh. |
| 15 | 13 or 14 |
| 16 | 12 not 15 |
| 17 | *Incidence/ |
| 18 | *Prevalence/ |
| 19 | (incidence or prevalence or epidemiol$).ti. |
| 20 | epidemiologic methods/ |
| 21 | *cohort studies/ |
| 22 | controlled clinical trial.pt. |
| 23 | *case-control studies/ |
| 24 | exp Anaphylaxis/ep [Epidemiology] |
| 25 | exp Hospitalization/ |
| 26 | exp Hospitalization/sn, td [Statistics & Numerical Data, Trends] |
| 27 | exp Mortality/sn, td [Statistics & Numerical Data, Trends] |
| 28 | exp Epinephrine/ad, tu, th [Administration & Dosage, Therapeutic Use, Therapy] |
| 29 | exp "Cause of Death"/ |
| 30 | ((adrenaline or epinephrine) adj3 (dispens$ or prescrib$)).tw. |
| 31 | or/17-30 |
| 32 | 16 and 31 |
| 33 | limit 32 to yr="1990 - 2012" |

*Database: Embase Classic+Embase <1990 to 2012 August 19>*

Search Strategy:

--------------------------------------------------------------------------------

| 1 | Anaphylaxis/ |
| --- | --- |
| 2 | anaphylaxis react*.mp. |
| 3 | anaphylactic react*.mp. |
| 4 | anaphylactic shock*.mp. |
| 5 | anaphylactoid syndrome*.mp. |
| 6 | anaphylactoid react*.mp. |
| 7 | anaphylactic syndrome*.mp. |
| 8 | anaphylactoid shock*.mp. |
| 9 | acute systemic allergic react*.mp. |
| 10 | idiopathic anaphylaxis.mp. |
| 11 | systemic anaphylaxis.mp. |
| 12 | or/1-11 |
| 13 | (rat or rats or cow or cows or chicken? or horse or horses or mice or mouse or bovine  or animal$).ti. |
| 14 | exp animals/ not humans.sh. |
| 15 | 13 or 14 |
| 16 | 12 not 15 |
| 17 | *Incidence/ |
| 18 | *Prevalence/ |
| 19 | (incidence or prevalence or epidemiol$).ti. |
| 20 | epidemiologic methods/ |
| 21 | *cohort studies/ |
| 22 | controlled clinical trial.pt. |
| 23 | *case-control studies/ |
| 24 | exp Anaphylaxis/ep [Epidemiology] |
| 25 | exp Hospitalization/ |
| 26 | exp Hospitalization/sn, td [Statistics & Numerical Data, Trends] |
| 27 | exp Mortality/sn, td [Statistics & Numerical Data, Trends] |
| 28 | exp Epinephrine/ad, tu, th [Administration & Dosage, Therapeutic Use, Therapy] |
| 29 | exp "Cause of Death"/ |
| 30 | ((adrenaline or epinephrine) adj3 (dispens$ or prescrib$)).tw. |
| 31 | or/17-30 |
| 32 | 16 and 31 |
| 33 | limit 32 to yr="1990 - 2012" |

*Database: CINAHL via Ebsco*

Search strategy:

-------------------------------------------------------------------------

| **#** | **Query** |
| --- | --- |
| S16 | S11 and S15 |
| S15 | S12 or S13 or S14 |
| S14 | (MM "Anaphylaxis") |
| S13 | "anaphylactic shock" |
| S12 | "anaphylactic" |
| S11 | S1 or S2 or S3 or S4 or S5 or S6 or S7 or S8 or S9 or S10 |
| S10 | (MM "Prevalence") |
| S9 | (MH "Incidence") |
| S8 | (MH "Prescribing Patterns") |
| S7 | "Epinephrine prescription" |
| S6 | "Epinephrine dispensing" |
| S5 | (MH "Epinephrine/AD/SD") |
| S4 | (MH "Epinephrine") |
| S3 | (MM "Hospitalization") |
| S2 | (MM "Disease Surveillance") |
| S1 | (MH "Epidemiology") OR (MH "Epidemiological Research") |

*Database: ISI Web of Science: Science Citation Index, Conference Proceedings Citation*

Search strategy:

| # 4 | #2 AND #1  Refined by: Web of Science Categories=( ALLERGY OR IMMUNOLOGY OR MEDICINE GENERAL INTERNAL ) AND Publication Years=( 1990-2012) AND Document Types=( PROCEEDINGS PAPER OR REVIEW OR MEETING ABSTRACT)  *Databases=SCI-EXPANDED, SSCI, A&HCI, CPCI-S, CPCI-SSH Timespan=All Years*  *Lemmatization=On* |
| --- | --- |
| # 3 | #2 AND #1  *Databases=SCI-EXPANDED, SSCI, A&HCI, CPCI-S, CPCI-SSH Timespan=All Years*  *Lemmatization=On* |
| # 2 | Topic=((epidemiol* or incidence or prevalance or surveillance or death or mortality or survival or prescrib* or prescript*))  *Databases=SCI-EXPANDED, SSCI, A&HCI, CPCI-S, CPCI-SSH Timespan=All Years*  *Lemmatization=On* |
| # 1 | Topic=(anaphylaxis or anaphylactic)  *Databases=SCI-EXPANDED, SSCI, A&HCI, CPCI-S, CPCI-SSH Timespan=All Years*  *Lemmatization=On* |
